# Age-associated changes in the growth development of abdominal fat and their correlations with cecal gut microbiota in broiler chickens

**DOI:** 10.1016/j.psj.2023.102900

**Published:** 2023-06-28

**Authors:** Xiaoying Liu, Chenxu Wang, Yumeng Wang, Chaohui Wang, Xi Sun, Yufei Zhu, Xiaojun Yang, Lixin Zhang, Yanli Liu

**Affiliations:** ⁎ College of Animal Science and Technology, Northwest A&F University, Yangling, China; † College of Life Sciences, Northwest A&F University, Yangling, China; ‡ Shanxi Dayu Biological Functions Co., Ltd., Yuncheng, Shanxi, China; § DAYU Bioengineering (Xi'an) Industrial Development Research Institute, Xi'an, Shaanxi, China

**Keywords:** abdominal fat, broiler chickens, cecal microbiota, development stage, 16S rRNA

## Abstract

Excess abdominal fat is a common phenomenon in broiler chickens. Gut microbiota could regulate lipid metabolism through their effects on short-chain fatty acids (**SCFAs**) production. This study was conducted to investigate the potential relationship between abdominal fat development and cecal microorganism populations. Abdominal fat and cecum contents were collected at 3, 7, 14, 21, 28, 35, and 42 d of age. The results showed that abdominal fat weight increased with age. The abdominal fat percentage was higher between 7 and 21 d of age than at 3 d (*P* < 0.05), and it increased again at 28 to 42 d (*P* < 0.05). Morphological analysis showed that adipocyte diameter and cross-sectional area (**CSA**) increased significantly after 14 d of age (*P* < 0.05). Moreover, gut microbiota analysis indicated that the Chao1 and Shannon indices were higher between 14 and 28 d than at 3 d of age (*P* < 0.05). Furthermore, LEfse analysis revealed that *Faecalibacterium, Anaerotruncus, Anaeroplasma, Subdoligranulum*, and *Clostridium* emerged to become dominant at 14 d. A greater abundance of *Bacteroides, Ruminococcus, Dehalobacterium*, and *Lactobacillus* were determined at 28 d when compared with 14 d of age. *Parabacteroides, Ochrobactrum, Lactobacillus, Blautia, Alistipes, Dehalobacterium, Odoribacter*, and *Suuterella* were found to be predominant at 42 d. PICRUSt analysis revealed that amino acid metabolism, lipid metabolism, and terpenoids and polyketides metabolism were elevated at 14 d; the immune and digestive systems were significantly developed at 28 d. In addition, cecum propionic acid and butyric acid contents gradually increased (*P* < 0.05), while the isobutyric acid contents gradually decreased with advancing age (*P* < 0.05). Correlation analysis among SCFAs, differential genera and abdominal fat suggested that *Coprobacillus, Shigella*, and *Butyricicoccus* had negative correlations with propionic acid, butyric acid, and abdominal fat weight, but positive correlations with isobutyric acid. Isobutyric acid was identified as being negatively associated with abdominal fat weight, while the reverse was found for propionic acid and butyric acid. In conclusion, abdominal fat development is correlated with the emergence of specific microbes and d 14 may be a pivotal age for establishing this relationship.

## INTRODUCTION

Improvements in broiler genetics have led to significant advances in growth rates and efficiencies as well as carcass yield, but one consequence is that in some cases fat accumulation can be excessive (Liang et al., 2022). Although abdominal fat is deposited later on in the life of the broiler (Liu et al., 2023), the rate of fat accumulation increases in older birds to the point where it exceeds other components and reduces efficiency and relative value (Wang et al., 2021). Therefore, attention needs to be focused on how to reduce abdominal fat accumulation. However little is understood about the development of the abdominal fat pad over the lifespan of the broiler, highlighting the need for further investigation into the mechanisms involved in controlling its accumulation.

The gut microbiota plays an important role in maintaining the health of the host (Zhou et al., 2022; Fan et al., 2023). It was reported that most chronic metabolic diseases were associated with the disturbance of gut microflora such as adiposity (Dabke et al., 2019), diabetes mellitus type 2 (Bouter et al., 2017), and nonalcoholic fatty liver disease (Lee et al., 2020). There is accumulating evidence in humans (Ley et al., 2005), mice (Ridaura et al., 2013), and livestock (Huang et al., 2020) suggesting that there is a strong relationship between gut microbiota and body fat mass. Transplanting the gut microbiota from obese donors to germ-free recipients significantly increased fat mass accumulation and metabolic syndrome (Wang et al., 2020a,b), and conversely a high-fat diet can lead not only to obesity but also to changes in the gut microbiota composition and function in mice (Cani et al., 2008; Wang et al., 2020a). *Akkermansia muciniphila* treatment can reverse high-fat diet-induced obesity, fasting blood glucose, and adipose tissue metabolism in mice (Everard et al., 2013). Excess abdominal fat deposition is usually associated with altered gut microbiota composition and metabolism functions (Ding et al., 2016; Wang et al., 2020a). Moreover, the gut microbiota was reported to be largely independent of host genetics in regulating fat deposition in chickens (Zhao et al., 2013). Wen et al. (2019) pointed out that the cecal microbiota had a significant effect on fat deposition and might account for 21% of the variance in the abdominal fat mass after correcting for host genetic effects via genome wide association study analysis. These points above imply that the gut microbiota is closely related to fat deposition in humans and animals.

Obesity, a systemic lipodystrophic syndrome, has become a common health problem throughout the world (Asadi et al., 2022). Similar to humans, the liver is mainly responsible for de novo lipogenesis in chickens and adipose tissue is the main site for lipid storage. However, both the liver and adipose tissue are considered to have equal importance for lipogenesis in rodents and rabbits. Therefore, the chicken is considered as a relevant biomedical model to study human obesity. However, few studies have focused on the relationship between abdominal fat and gut microbiota in chickens. The current study was conducted to analyze age-associated changes in the development of abdominal fat and further reveal their correlations with cecal gut microbiota in broiler chickens. The aim of this work was to provide guidance on whether nutritional strategies that alter fat deposition may be a result of their effects on the gut microbiota.

## MATERIALS AND METHODS

All broiler chickens and experimental protocols in the study were approved by the Institution Animal Care and Use Committee of Northwest A&F University (Permit Number: DK202123).

### Animals and Sample Collection

All broiler chickens were obtained from the Xian Dacheng Poultry Industry Co., Ltd. (Xianyang, China). A total of 300 one-day-old Arbor Acres broiler chickens were raised with free access to a commercial diet and water at the Experimental Teaching Center of Animal Science at the Norwest A&F University. At the age of 3, 7, 14, 21, 28, 35, and 42 d, 12 birds (half male and half female) were selected and killed by cervical dislocation and dissected, and then abdominal fat was removed and weighed. The abdominal fat percentage was expressed relative to body weight (g of organ/g of body weight). Fresh cecum chymus was collected for subsequent gut microbiota and short-chain fatty acids (**SCFAs**) analysis. After sampling, abdominal fat and cecum chymus were immediately frozen in liquid nitrogen and stored at −80°C.

### Abdominal Fat Morphology Analysis

Approximately 1 cm^3^ abdominal fat tissue pieces were fixed in 4% formaldehyde for more than 48 h for hematoxylin and eosin (**H&E**) histological analysis, which was performed by Wuhan Servicebio Technology Co., Ltd. (Wuhan, China). Abdominal adipose tissue sections were examined and photographed using an inverted fluorescence microscope, and the diameter and area of adipocytes were determined using Image-Pro Plus 6.0 software (Media Cybernetics, Silver Spring, MD) based on the previously reported method (Chen and Farese, 2002).

### Microbiota DNA Extraction and 16S Library Construction

Microbiota genomic DNA of cecal contents was extracted for quality detection by agarose gel electrophoresis and UV spectrophotometer and bacterial V3V4 regions were amplified using the following primers: F: ACTCCTACGGGAGGCAGCA, R: GGACTACHVGGGTWTCTAAT. The PCR amplification program was as follows: denaturation at 94°C to 96°C for 1 min, annealing at 50°C to 60°C for 30 s, and extension at 72°C for 1 min, repeated for 35 cycles. After amplification, PCR product purification was performed using the AxyPrep DNA Gel Extraction Kit (Axygen, Phoenix, AZ), then purified DNA was quantified for library construction and sequencing on the Illumina platform, which was carried out by Shanghai Personal Biotechnology Co., Ltd., Shanghai, China.

### Bacterial Community Composition and Diversity Analysis

After filtering, merging, removing impurities, and pruning effective sequences, sequencing data were compared using the Greengene database to analyze alpha- and beta-diversity and detect the relative abundance at the phylum, class, order, family, and genus levels according to QIIME2 software (http://qiime.org/index.html). Furthermore, LEfSe analysis was applied to determine differential microbiota among different days based on the following parameters: linear discriminant analysis score >2 and *P* < 0.05.

### Functional Prediction Analysis

Phylogenetic investigation of communities by reconstruction of unobserved states (**PICRUSt**) was employed to predict microbial functional changes (Langille et al., 2013). The sequencing information was automatically normalized to known bacterial genomes from the Integrated Microbial Genomes database. Predicted functions were performed with the Kyoto Encyclopedia of Genes and Genomes (**KEGG**) database based on 16S rRNA genes, and STAMP software was used to analyze functional differences along with the age in broiler chickens.

### Short-Chain Fatty Acids

Cecal acetic acid, propionic acid, butyric, acid and isobutyric acid contents were detected based on our previous report (Liu et al., 2023). Briefly, cecal chymus was homogenized in cold normal saline. Then the supernatant was collected and mixed with metaphosphoric acid and crotonic acid sequentially following centrifugation at 10,000 × *g* for 15 min. After pretreatment, samples were analyzed using the GC-MS method via the ratio of the peak area with the standard solution.

### Statistical Analysis

All experimental data were analyzed by Statistical software SPSS 23.0 (SPSS Inc., Chicago, IL) and plotted by GraphPad Prism 8. The test data were expressed as the mean with standard error and analyzed by the 1-way ANOVA, and Duncan's multiple comparisons test was used to determine significance. A probability value less than 0.05 was considered statistically significant.

## RESULTS

### Growth Performance

**Figure 1:**
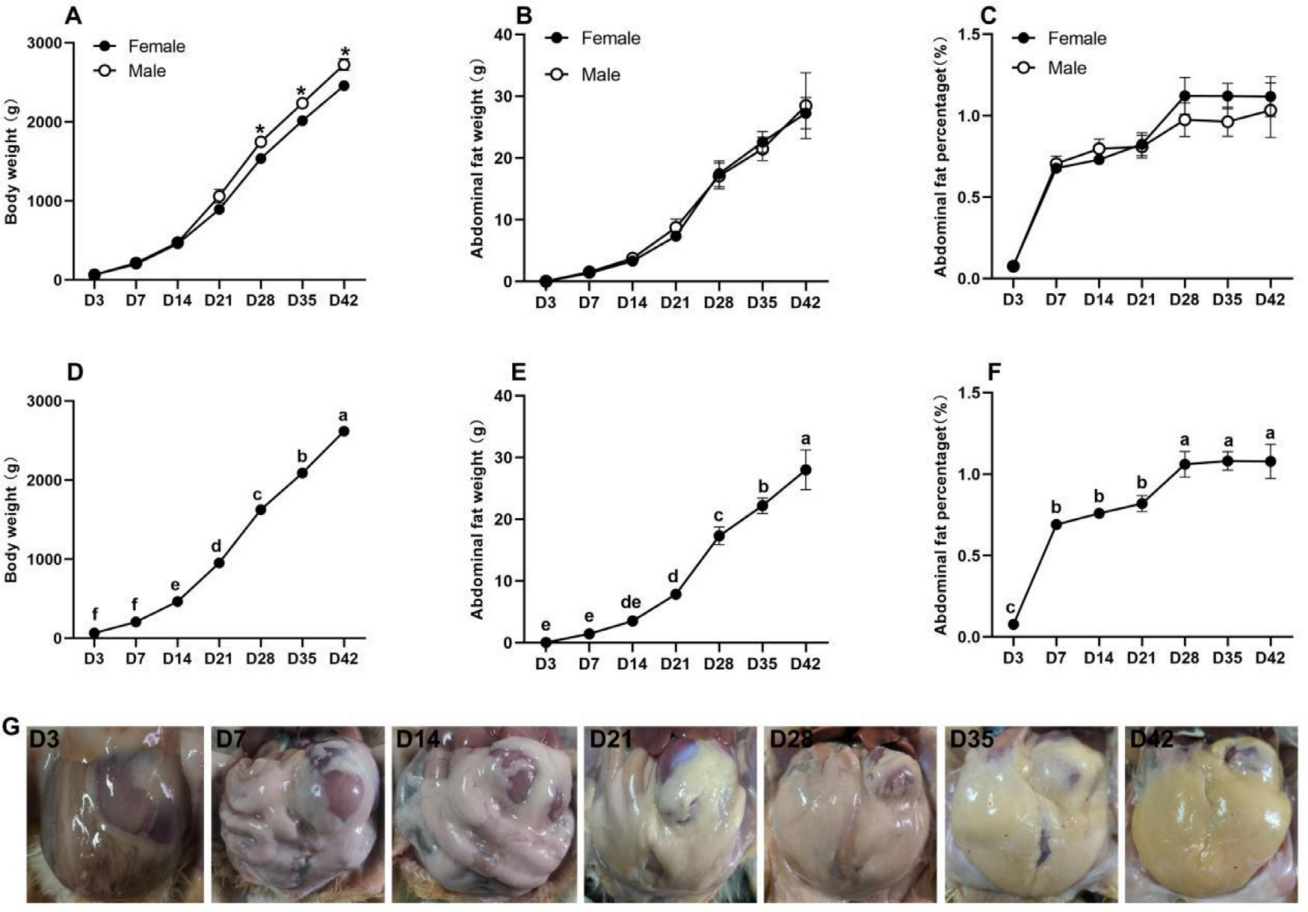
The observation of abdominal fat phenotypes. (A–C) Body weight, abdominal weight, and abdominal percentage for male or female broiler chickens. Data are expressed as mean with standard error, and the asterisk (*) indicated statistically significant differences (2-tailed unpaired test, *P < 0.05). (D and E) Body weight, abdominal weight, and abdominal percentage in broiler chickens without considering genders. Data are expressed as mean with standard error, and the different letters indicate significant differences and the same letter indicates no significant differences (1-way ANOVA, *P* < 0.05). (F) Abdominal fat anatomy observation.

As shown in Figure 1A to C, there was no difference in abdominal fat weight and abdominal fat percentage between male and female chicks at the same age. However, body weight in male birds was higher than females during 28 to 42 d. Figure 1D and E showed that the body weight and abdominal fat weight increased gradually from D3 to D42 for both sexes. The abdominal fat percentage increased significantly from 7 d of age (*P* < 0.05), and remained at approximately 0.75% until 21 d, and then increased again to approximately 1% from 28 to 42 d as shown in Figure 1F (*P* < 0.05). As shown in Figure 1G, there was little abdominal fat deposition at D3 but this increased significantly with age.

### Abdominal Fat Morphology Analysis

**Figure 2:**
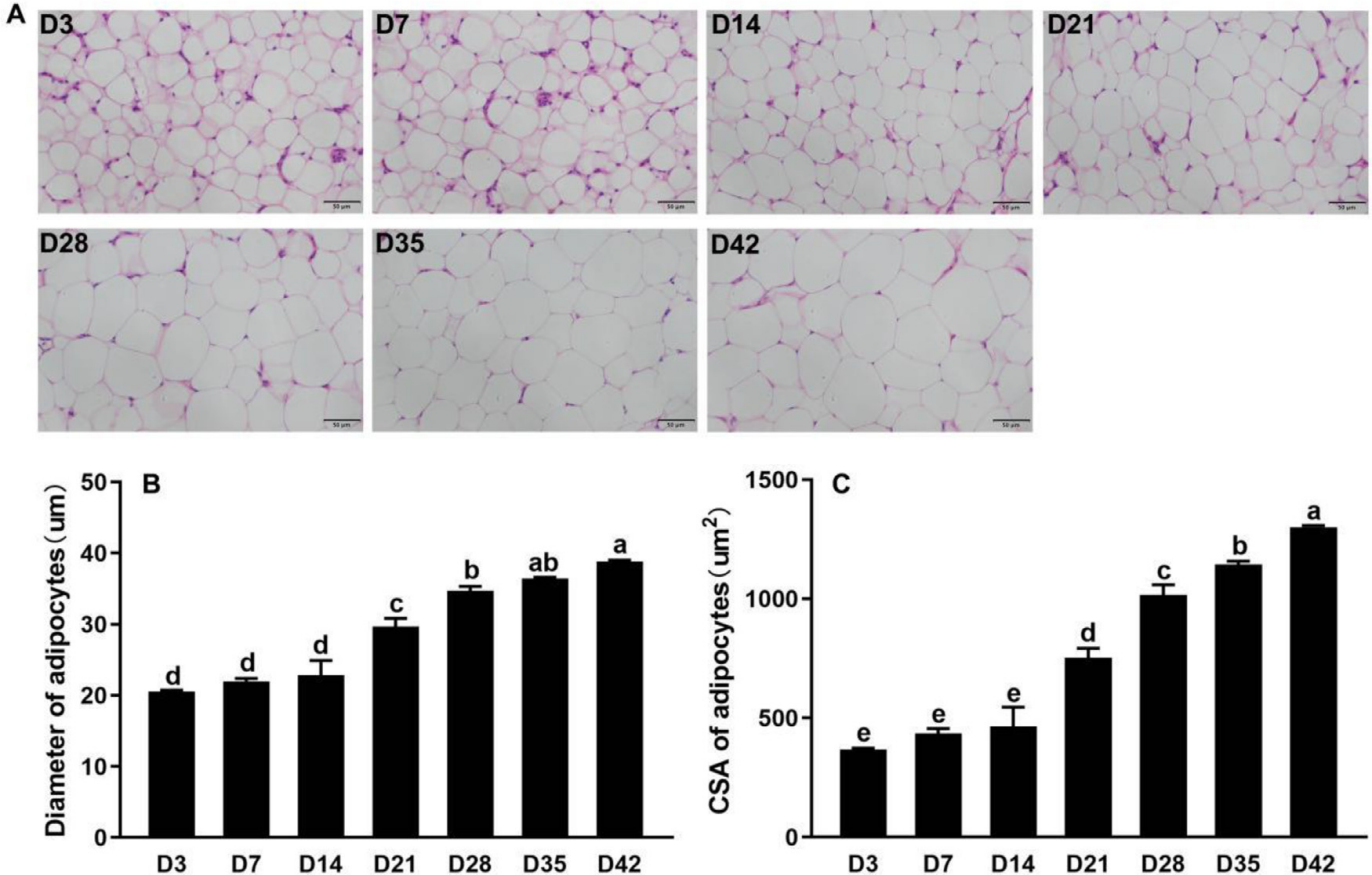
Morphological analysis, adipocyte diameter, and area statistics for abdominal fat. (A) H&E staining for abdominal fat tissue (magnification: 10 × 40; the scale is 50 um). (B) and (C) adipocyte diameter and CSA statistics. The different letters indicate significant differences and the same letter indicates no significant differences (1-way ANOVA, *P* < 0.05).

To investigate the dynamic changes of abdominal adipocytes during the whole life of broiler chickens we performed morphology analysis for abdominal adipose tissue at D3, D7, D14, D21, D28, D35, and D42. As displayed in Figure 2A, the adipocytes looked smaller at D3, and enlarged with age. The proportion of cells with a diameter of 10 to 20 um decreased gradually while for cells with a diameter of more than 30 um the proportion increased (Figures S1 and S2). The results in Figure 2B and C showed that the average diameter and cross-sectional area (**CSA**) of adipocytes increased significantly after D14 (*P* < 0.05).

### Cecum Microbiota

**Figure 3:**
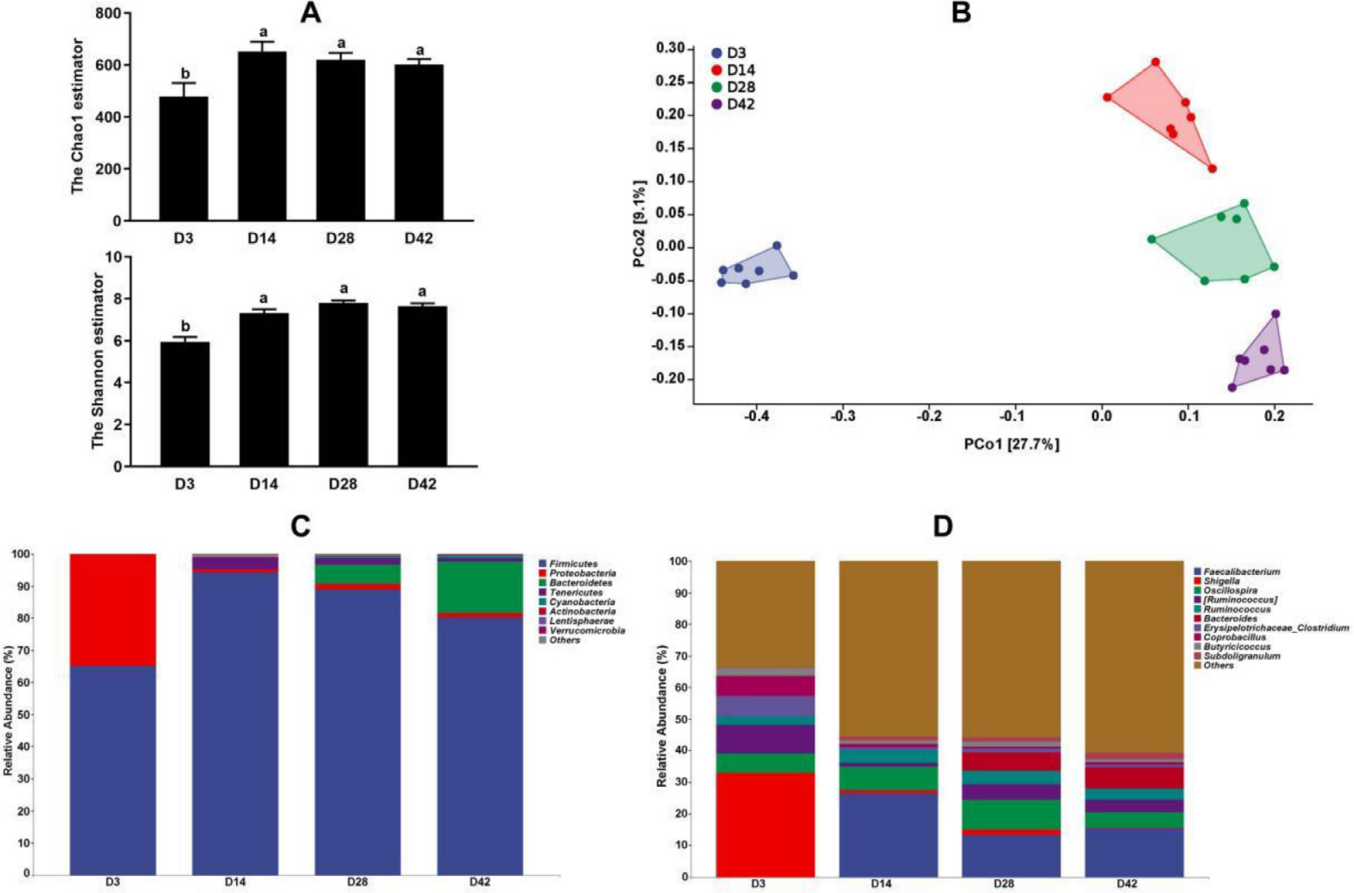
Cecum microbiota analysis in broiler chickens. (A) Chao1 and Shannon index. (B) Principal coordinates analysis (PCoA). (C and D) Relative abundance of bacterial composition at phylum level and genus level. Every color means 1 bacteria and *Y*-axis showed the relative abundance of the bacteria.

**Figure 4:**
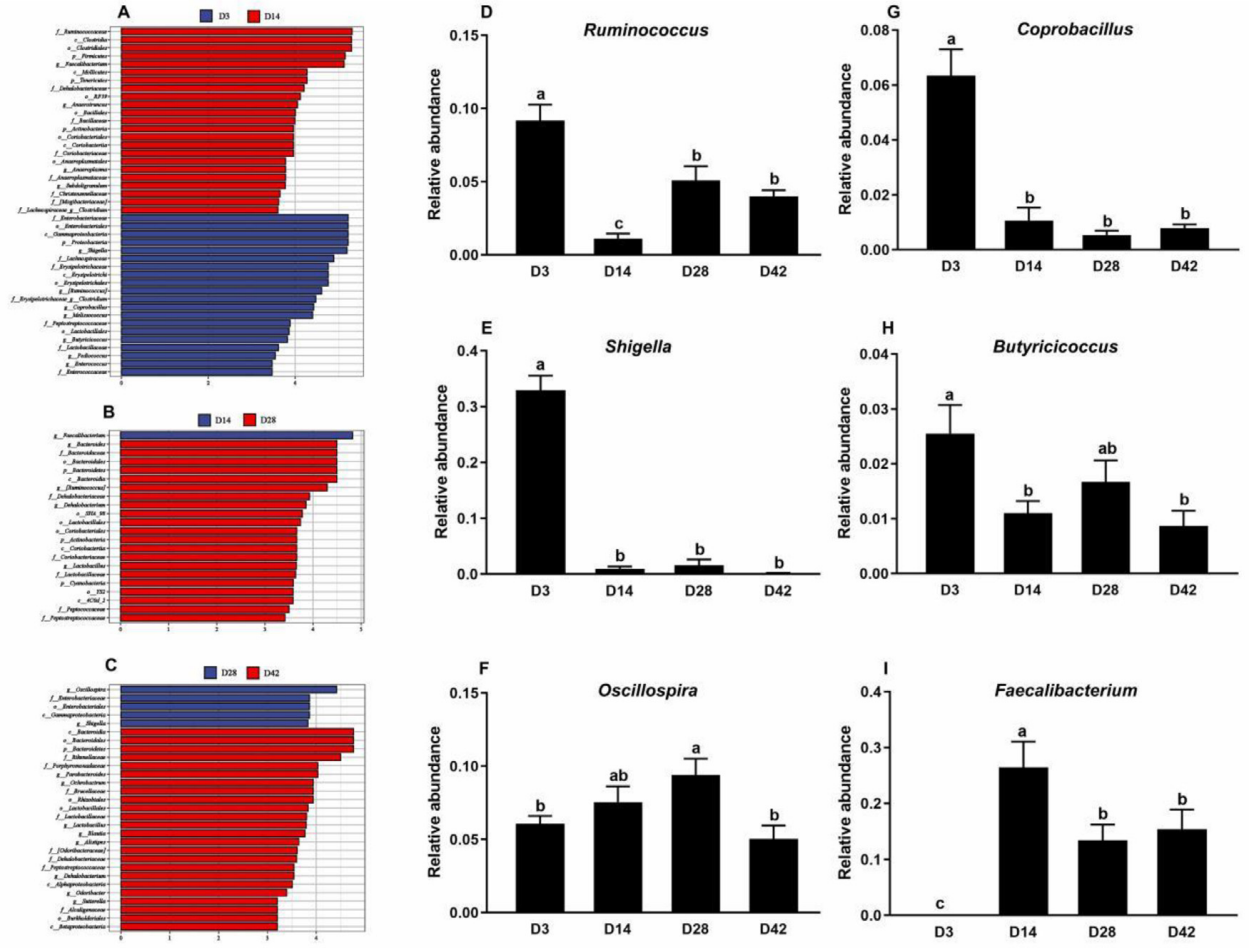
Differential microbiota analysis. (A–C) Differential microbiota based on LEfSe method between 2 adjacent stages. The default parameters were LDA score >2 and *P* < 0.05. (D–I) Relative abundance analysis for selected differential bacteria. Data are expressed as mean with standard error (*n* = 7). The different letters indicate significant differences and the same letter indicates no significant differences (1-way ANOVA, *P* < 0.05).

**Figure 5:**
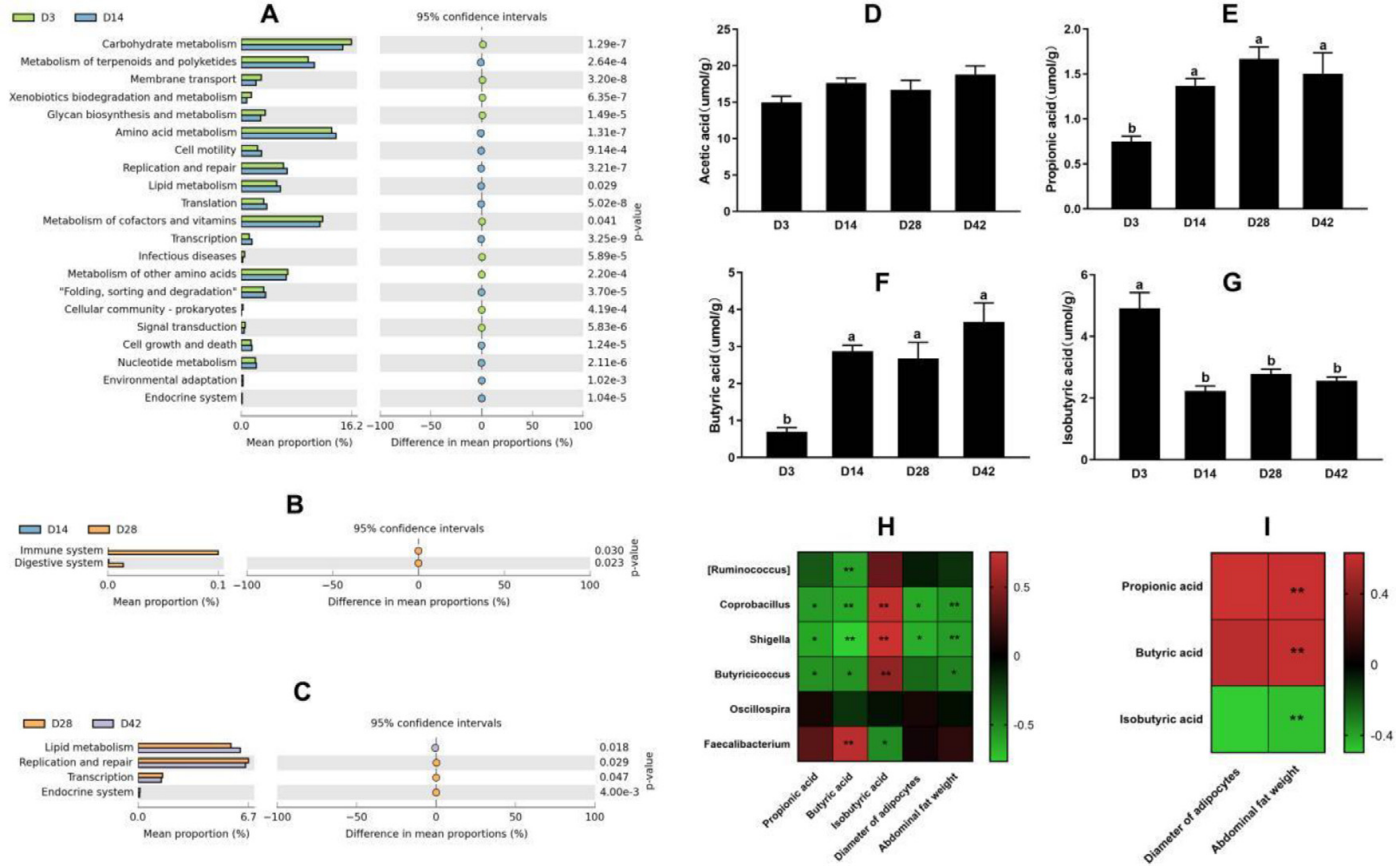
Functional predicted analysis of cecal microbiota, cecum SCFAs, and their correlation analysis. (A–C) Comparisons of functional pathways. (D–G) Cecum SCFA concentrations of broiler chickens. Data are expressed as mean with standard error (*n* = 7). Different letter indicates significant correlation (*P* < 0.05), the same letter or no letter indicates no significance (*P* > 0.05). (H and I) Heatmap of correlation analysis among cecal microbiota, cecum SCFAs, diameter of adipocytes, and abdominal fat weight. * or ** means that there is a significant correlation between 2 indices at the 0.05 or 0.01 level (2-tailed). Red and green colors represent positive and negative correlation, respectively, and color gradation indicates the size of the correlation coefficient.

Considering the results of abdominal fat weight and adipocyte diameter in broiler chickens, we selected 4 stages (D3, D14, D28, and D42) for gut microbiota analysis. As shown in Figure 3A, the alpha-diversity indices including Chao1 and Shannon were higher at D14, D28, and D42 than at D3. Principal coordinates analysis (**PCoA**) presented significant differences and separation for microbial communities among 4 different days (Figure 3B). *Fimicutes, Proteobacteria, Bacteroidetes*, and *Tenericutes* are the most abundant phyla in broiler chickens. *Proteobacteria* was higher at D3 and decreased along with the age, while *Bacteroidetes* apparently increased from D28 (Figure 3C). At the genus level, the relative abundance of *Shigella* accounted for about 30% at D3 but decreased significantly from D14 (Figure 3D). Further, LEfSe analysis was used to identify dominant microbiota between 2 adjacent days (Figure 4A–C) and results showed the following: the dominant microflora were *Faecalibacterium, Anaerotruncus, Anaeroplasma, Subdoligranulum*, and *Clostridium* at 14 d when compared with those at 3 d; the higher abundance of *Bacteroides, Ruminococcus, Dehalobacterium*, and *Lactobacillus* were determined at 28 d in comparison with 14 d; *Parabacteroides, Ochrobactrum, Lactobacillus, Blautia, Alistipes, Dehalobacterium, Odoribacter*, and *Suuterella* were found to be predominant at 42 d. Meanwhile, as shown in Figure 5D to I, with the advancing age, the relative abundances of *Ruminococcus, Coprobacillus, Shigella*, and *Butyricicoccus* decreased when compared with those at D3, while *Oscillospira* and *Faecalibacterium* were found to increase first and then decrease from D42 and D28, respectively.

### Functional Prediction Analysis for Cecal Microbiota

The functional prediction for the cecal microbiota between 2 adjacent developmental stages was performed using PICRUSt method. As shown in Figure 5A to C, functional capacities involved in carbohydrate metabolism, glycan biosynthesis, and metabolism, and metabolism of cofactors and vitamins were enriched significantly at D3. Amino acid metabolism, lipid metabolism, terpenoids, and polyketides metabolism were enhanced at D14 when compared with D3. Furthermore, the immune and digestive systems were significantly developed at D28, and the gut microbiota were predicted to have a significant involvement in lipid metabolism again at D42.

### Cecum SCFAs and Their Correlation Analysis

As displayed in Figure 5D to G, cecal acetic acid content did not change with time. However, propionic acid and butyric acid concentration increased from D14 (*P* < 0.05) and reached an asymptote between 14 and 42 d, while the inverse effect was noted for isobutyric acid. Pearson correlation analysis among cecal microbiota, SCFAs, and abdominal fat phenotypes was carried out and results were shown in Figure 5H and I. The relative abundances of *Coprobacillus, Shigella*, and *Butyricicoccus* were significantly negatively correlated with propionic acid, butyric acid, and abdominal fat weight, but positively correlated with isobutyric acid. There was a negative relationship between bacteria (*Coprobacillus* and *Shigella*) and adipocyte diameter. Isobutyric acid was negatively correlated with abdominal fat weight, while propionic acid and butyric acid were positively related to abdominal fat weight.

## DISCUSSION

Excess abdominal fat deposition is a common issue in broilers, which not only reduces feed utilization efficiency but also adversely affects host health. Recently, numerous studies have reported that gut microbes are inextricably linked to fat deposition (Everard et al., 2013; Anto and Blesso, 2022). Our previous study indicated that dietary folic acid could reduce abdominal fat deposition by altering gut microbiota and SCFAs in broiler chickens (Liu et al., 2023). Cecal microbiota was considered to be involved in nonalcoholic steatohepatitis in laying hens (Hamid et al., 2019). These results suggest that there is a relationship between abdominal fat accumulation and the development of the cecal microbiota. This work was therefore initiated to determine if such a correlation exists between specific bacteria and abdominal fat pad accumulation.

Adipose tissue is the main organ for fat storage in poultry. The number and size of adipocytes are closely related to fat synthesis and deposition (Wang et al., 2017). The morphological study on abdominal adipose tissue found that the number and size of adipocytes increased with age in high-fat and low-fat line chickens (Guo et al., 2011), which was consistent with our results that adipocyte diameter and area increased along with the age of broiler chickens from D14. In the current study, abdominal fat weight gradually increased between 3 and 14 d of age, but there was no difference in the average diameter and mean area of adipocytes in this period which implied that adipocyte proliferation (i.e., number) might contribute more to the increase in abdominal fat weight during this period. On the other hand, the percentage of adipocytes with 30 to 50 um diameter started to increase at D14 onward while cells between 10 and 20 um diameter decreased in proportion. In addition, abdominal fat weight reached a significant inflection point at D14. Thus D14 might be a key physiological stage for abdominal adipocyte hypertrophy. This also explains the accelerated increase in abdominal fat weight between 14 and 42 d, which might be due not only to adipocyte proliferation but also to cell differentiation. A previous study also reported that genes involved in adipocyte differentiation were more highly expressed at D14 than at D4 such as PPARγ, FABP4, and LPL (Bai et al., 2015), indicating that cell differentiation indeed took place in abdominal fat of broiler chickens at D14.

Huang et al. (2018) reported that the gut microbiota developed into a relatively mature community around 28 d of age in broiler chickens based on the metagenome analysis. Considering the physiological characteristics of fat deposition, we selected 4 stages (D3, D14, D28, and D42) for further gut microbiota analysis. Our results showed that more abundant cecal bacterial communities were determined in broiler chickens from 28 d of age. PCoA analysis revealed more similarity for cecum microbiota composition between 14, 28, and 42 d, indicating that the establishment and development of the cecal microbiota in broiler chickens was mainly completed between 14 and 28 d. These findings were in accordance with a previous study in which the gut microbiota tended to be stable between 14 and 28 d in chickens (Ballou et al., 2016). Further, function prediction of gut microbiota revealed that more complex metabolic functions such as amino acid and lipid metabolism were enriched at D14 as well as that for terpenoid and polyketide metabolism. Moreover, the immune and digestive system were evolved up to D28, suggesting cecal microbiota could take part in regulating immune and digestive function of hosts based on the mature of structure and function on gut microbiota. These results implied that gut microbiota reached maturity at approximately D28 in broiler chickens, which is also supported by the SCFAs results in the current study because there was no difference in acetic acid, propionic acid, butyric acid, and isobutyric acid concentration in cecum at D28 when compared with D14 or D42.

Cornejo-Pareja et al. (2019) noted that fat deposition was related to changes in microbial community composition. It was reported that the relative abundance of *Firmicutes* is higher in the gut of obese animals, while the relative abundance of *Bacteroidetes* is higher in lean individuals (Walters et al., 2014). *Firmicute*s was considered to be positively correlated with fat deposition in animals in a separate study (Patterson et al., 2016). To reveal the potential genus associated with fat deposition in the life of the broiler, different bacterial genera with relatively higher abundance were picked and used to perform correlation analysis together with abdominal fat phenotypes. The results showed that *Coprobacillus* and *Shigella* were identified as being negatively associated with the adipocyte diameter and abdominal fat weight. The same phenomenon was found between abdominal fat weight and *Butyricicoccus*. These results imply that these 3 genera might regulate abdominal lipid deposition. In addition, the lipid metabolism pathway was significantly enriched at both D14 and D42 based on functional predictions of gut microbiota, suggesting the cecal microbiota might indeed play a key role in fat deposition in broiler chickens.

SCFAs are fermentation products from intestinal microorganisms metabolizing dietary nutrients, which bridges gut microbiota and host physiology. It was reported that SCFAs could promote energy metabolism and fatty acid synthesis, thus affecting the accumulation of body fat (Cronin et al., 2021). In the current study, the cecum contents of propionic acid and butyric acid gradually increased with the advancing age, while the opposite result was found for isobutyric acid. Correlation analysis was performed to reveal the relationship between SCFAs and differential genera as well as abdominal fat phenotypes. Propionic acid and butyric acid were positively related to abdominal fat weight. It was possible that increased propionic acid and butyric acid provided more energy for adipocyte hyperplasia and hypertrophy. Recent studies have found that propionic acid can promote the increase of intracellular fat content in mice preadipocytes (Yeganeh et al., 2016; Wang et al., 2020a). It was reported that butyric acid could regulate the acetylation level of lipid-related genes by inhibiting histone deacetylase (**HDAC**) activity, thereby affecting fat synthesis and deposition (Yan and Ajuwon, 2015; Huang et al., 2017). However, as an important butyric acid-producing bacteria, *Butyricicoccus* was found to be positively associated with isobutyric acid rather than butyric acid. In addition, isobutyric acid was identified as being negatively correlated with abdominal fat weight, indicating that *Butyricicoccus* might be a potential bacterium for reducing fat deposition. The same relationship was found between *Coprobacillus* and *Shigella* and isobutyric acid. Thus, isobutyric acid might be a marker reflecting abdominal fat deposition in broiler chickens.

## CONCLUSIONS

In conclusion, the current study revealed age-associated changes in the development of abdominal fat and their correlations with cecal gut microbiota in broiler chickens. Abdominal fat development is correlated with the emergence of specific microbes and d 14 may be a pivotal age for establishing this relationship. On the other hand, *Coprobacillus, Shigella*, and *Butyricoccus* were identified to be related to abdominal fat deposition via regulating isobutyric acid production, which needs to be validated in the future.

## Appendix Supplementary materials

Image, application 1

## Footnotes

Supplementary material associated with this article can be found in the online version at doi:10.1016/j.psj.2023.102900.
