# Supplementary material for: Age-associated changes in the growth development of abdominal fat and their correlations with cecal gut microbiota in broiler chickens
Source: Poult Sci. 2023 Jun 28;102(9):102900. doi: 10.1016/j.psj.2023.102900 (PMC10466292; doi:10.1016/j.psj.2023.102900)
Supplement: Supplementary file 1 [file mmc1.docx]

**Supplemental Figures**


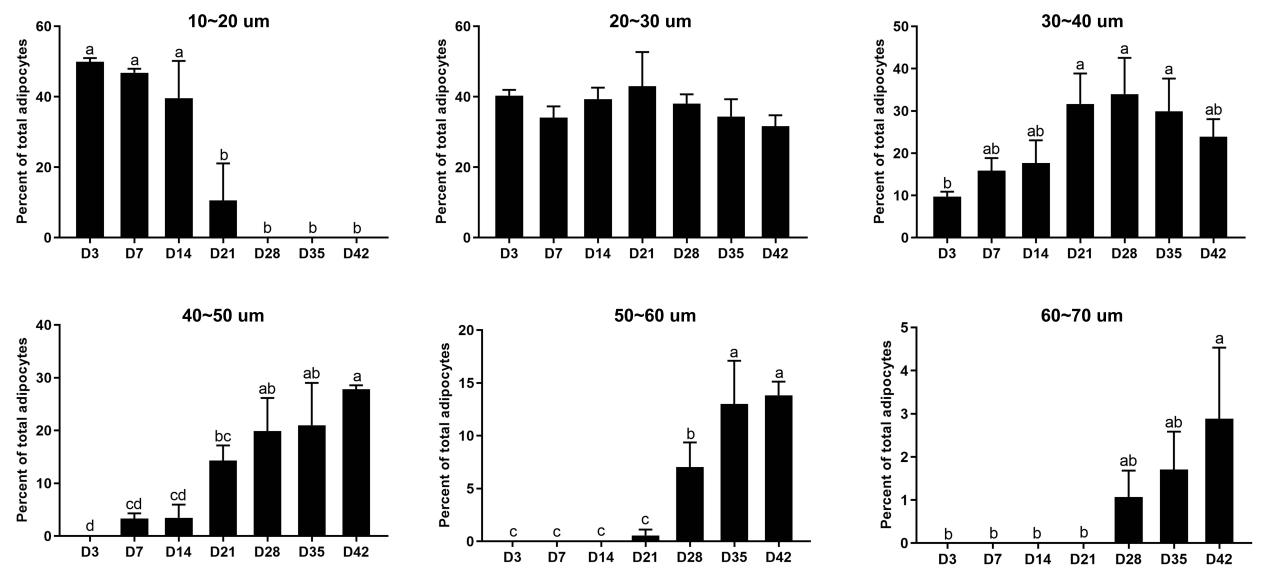


**Figure S1** Adipocyte diameter distribution in broiler chickens. Results are given as mean with standard error for 3 independent sections of 4 chickens for each group. Cells with diameters less than 10 um were not included.


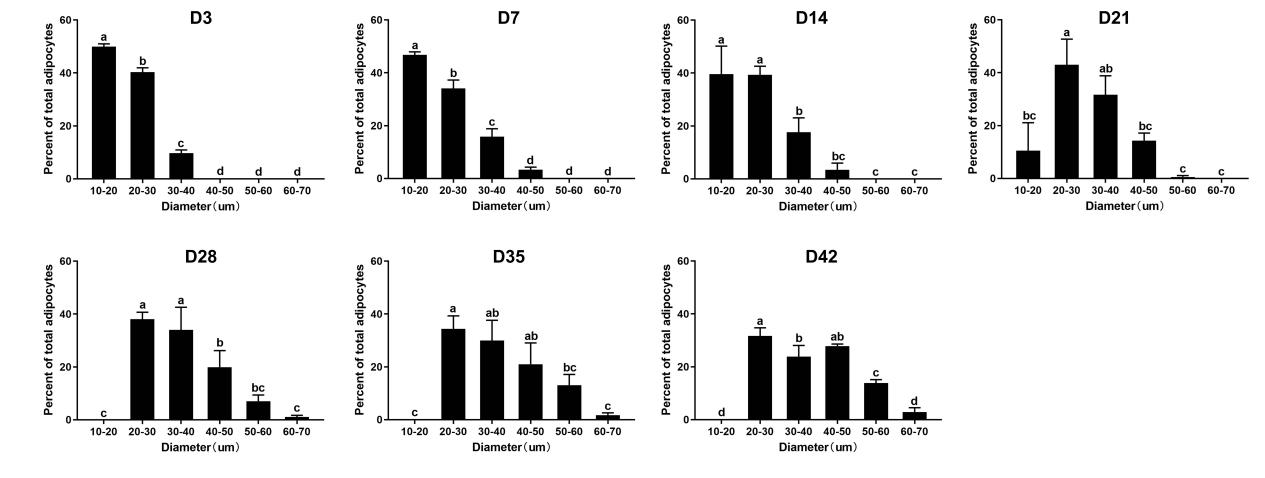
**Figure S2** Changes of adipocyte diameter in broiler chickens. Results are given as mean with standard error for 3 independent sections of 4 chickens for each group. Cells with diameters less than 10 um were not included.

**Supplemental Table**

Table S1 Formulation and proximate composition of experimental diets

| Items | Values | |
| --- | --- | --- |
|  | 1 to 21 days | 22 to 42 days |
| Ingredients, % |  |  |
| Corn | 57.27 | 60.99 |
| Soybean meal | 35.20 | 30.00 |
| Cottonseed mea | 2.00 | 4.00 |
| Soybean oil | 2.00 | 2.00 |
| NaCl | 0.36 | 0.35 |
| Limestonel | 2.00 | 1.70 |
| Calcium hydrogen phosphate | 0.30 | 0.30 |
| Choline chloride | 0.05 | 0.05 |
| *L*-lysine hydrochloride | 0.14 | 0.06 |
| Mineral premix^a^ | 0.30 | 0.30 |
| Phytase | 0.10 | 0.10 |
| Vitamin premix^b^ | 0.03 | 0.03 |
| DL-methionine | 0.25 | 0.12 |
| Total | 100.00 | 100.00 |
| Nutrient levels^c^, % |  |  |
| apparent metabolizable energy, Kcal/kg | 2949.26 | 3044.86 |
| Crude Protein | 21.91 | 19.89 |
| Calcium | 0.96 | 0.91 |
| Total phosphorus | 0.61 | 0.60 |
| Available phosphoruo | 0.40 | 0.40 |
| Methionine | 0.57 | 0.45 |
| Methionine+Cysteine | 0.90 | 0.75 |
| Lysine | 1.21 | 1.05 |

^a^Mineral premix provided the following per kg of the diet: Mn, 80 mg; I, 0.40 mg; Fe, 80 mg; Cu, 10 mg; Zn, 70 mg; Se, 0.30 mg.

^b^Vitamin premix provided the following per kg of the diet: vitamin A, 250,000 IU; vitamin D, 50,000 IU; vitamin K3, 53 mg; vitamin B1, 40 mg; vitamin B2, 120 mg; vitamin B12, 0.50 mg; vitamin E, 600 IU; biotin, 0.65 mg; folic acid, 25 mg; pantothenic acid, 240 mg; niacin, 1,000 mg.

^c^The nutrient levels were calculated values.
